# Qualitative research to explore the symptoms and impacts experienced by children with ulcerative colitis

**DOI:** 10.1186/s41687-020-00238-1

**Published:** 2020-09-07

**Authors:** Jason A. Randall, Aiste Guobyte, Laure Delbecque, Louise Newton, Tara Symonds, Theresa Hunter

**Affiliations:** 1 Clinical Outcomes Solutions, Unit 68 Basepoint, Shearway Business Park, Shearway Road, Folkestone, Kent CT19 4RH UK; 2 Clinical Outcomes Solutions, Chicago, IL USA; 3 Eli Lilly and Company, Brussels, Belgium; 4 Eli Lilly and Company, Indianapolis, IN USA

**Keywords:** Ulcerative colitis, Children, Parents, Caregiver, Qualitative, Experience

## Abstract

**Background:**

Ulcerative Colitis (UC) is a chronic gastrointestinal disease that often presents during one’s most productive years and is characterized by colon inflammation. Key symptoms and impacts in adults are well-known, however, experiences among pediatric populations have not been well documented. The purpose of this study was to understand the health-related quality of life and symptomatic experience of children (2–11 years) living with UC.

**Methods:**

Qualitative, semi-structured face-to-face interviews were conducted. Children aged 5–11 years were interviewed, as well as their parents/caregivers in matched dyads. Parents/caregivers of children aged2–4 years were interviewed within a parent/caregiver-only cohort. All participants were recruited from the United States. Interviews were coded using thematic analysis.

**Results:**

Key symptoms and impacts reflecting the lived experience of UC were identified following thematic analysis, generating a conceptual model. A total of 32 participants (20 parents/caregivers and 12 children) were interviewed. Results identified a substantial burden of UC in children. All children and parents/caregivers reported that they/their child experienced stomach/abdominal pain. Other symptoms discussed by over 75% were blood in stool, diarrhea/loose stools, stool urgency, incomplete evacuation, stool frequency, and feeling gassy/passing gas. The most frequently discussed impacts by over 75% of participants were on emotional and practical aspects, seriously affecting quality of life.

**Conclusions:**

Qualitative analysis of the interviews identified a substantial burden of UC on children, with a profound impact on their lives. The symptomatic experience is reflective of adults and adolescents. A high level of agreement between parents/caregivers and children was demonstrated regarding the perception of the presence or absence of symptoms. Children aged 8–11 years showed higher levels of agreement with parents/caregivers than did younger children, indicating appropriateness of self-report of symptom data in the 8–11 years age group.

## Background

Ulcerative Colitis (UC) is a chronic gastrointestinal disease of unknown cause characterized by inflammation in the colon [1, 2]. UC often presents in adolescence and early adulthood, but can also present in childhood [1]. The incidence of UC is 9 to 20 cases per 100,000 persons per year and the prevalence is 156 to 291 cases per 100,000 persons per year [3].

Patients diagnosed with UC in childhood typically have more extensive disease, with more frequent, acute and severe exacerbations when compared to those diagnosed as adults [1]. There has been debate as to whether, across the age spectrum, we see consistent features of the same disease [4] and this research explores this question of continuum. Regardless of age, we know that those with UC and their parents/caregivers report intermittent disease flares interspersed with periods of remission [1, 2].

There is limited qualitative information available on the symptoms and health-related quality of life (HRQoL) impacts reported by patients with UC. Most of the qualitative articles available in the literature do not focus explicitly on a UC population. Rather they include patients with Crohn’s disease or other similar nonspecified inflammatory bowel disease conditions. Most articles focus on adult-only populations with mixed etiologies, but provide an initial perspective for the symptoms and HRQoL experiences of patients with UC [5–22].

More recently, a qualitative study of adults and adolescents with UC was undertaken [23] to explore their symptomatic and HRQoL experience. This resulted in the generation of a UC conceptual disease model, key outcomes of interest in this population, and the hypothesized relationship between outcomes, which may inform decision-making. This study established that the symptomatic and HRQoL journey between adolescents and adults were not attributable to age-specific populations. This study showed that the most important symptoms experienced by ≥75% of participants (adults and adolescents) were stomach/abdominal pain, frequent bowel movements, diarrhea, blood in stools, sudden need for bowel movement, stomach cramping, bloating, and feeling gassy/passing gas. Whilst the most important (≥ 75% of participants) HRQoL impacts were embarrassment, dietary limitations, having to plan around UC, worry/fear, anger, low mood/depression, and relationship with others.

The United States (US) Food and Drug Administration’s (FDA) 2009 guidance on the use of patient-reported outcome (PRO) measures in medical product development [24], highlights the need to elicit direct patient input as part of early qualitative research to enable identification of all relevant concepts to be assessed in future clinical trials. This guidance is supported by the US Twenty-first Century Cures Act [25], which was designed to help accelerate medical product development and bring new innovations and advances to patients who need them faster and more efficiently. The Act highlights that the lived experience of the patient should be incorporated into drug development where possible. As a consequence, a recent draft guidance from the FDA [26] indicated that more research is needed in UC, particularly pediatric populations, to understand the disease burden of this condition and help identify the optimum measurement strategy.

This research, therefore, arises partly from these recommendations from the FDA to further study pediatric UC populations (aged 2–11 years) [26]. Previous work has established continuity and homogeneity between the experiences of adolescents and adults with UC [23]. A better understanding of disease symptomatology, impact across the age spectrum, establishing or dismissing continuity and homogeneity of experience, and disease burden from childhood to adulthood will be of value. The aim of this study is to explore the lived experience of UC from a child’s (2–11 years) perspective and to better understand how symptoms impact children’s lives, both generally and during disease flares.

## Methods

### Study design and participants

Individual face-to-face qualitative interviews were conducted with children aged 5–11 years who had a clinical diagnosis of UC and with their parents/caregivers. Parents/caregivers of UC diagnosed children aged 2–4 years were also interviewed. All interviews were conducted in the US. The study was designed from a phenomonelogical perspective, ie, the aim was to generate knowledge about how individuals experience things, with a focus on understanding the ‘lived experience’ of UC [27].

### Recruitment and sampling

Participants were recruited from 5 sites (Chicago IL, New Orleans LA, Knightdale NC, Greenville SC, and Braintree, MA) in the US using referrals from clinicians. These clinicians reviewed their database to identify potential participants meeting the eligibility criteria for this study using a Health Insurance Portability and Accountability Act waiver. Children aged 5–11 years and their parent/caregiver were interviewed separately as part of a matched dyad; for children aged 2–4 years, only the parent/caregiver was interviewed.

Participants were eligible for inclusion if they were aged 5–11 years with a diagnosis of UC, or were parents/caregivers of children aged 2–11 years with a diagnosis of UC, and fluent in age-appropriate US English. Diagnosis of UC had to be confirmed by sigmoidoscopy or colonoscopy at least 3 months prior to the interview to ensure participants had disease experience. Child participants had to experience mild to severe UC over the past 30 days as confirmed by his or her clinician.

Participants were excluded if a surgical resection had significantly treated the UC or if they had a previous diagnosis of Crohn’s disease, indeterminate colitis, radiation colitis, or diverticular-associated colitis that might interfere with the participant’s ability to evaluate the symptoms and impacts of their (or their child’s) UC. In addition, those with a significant or uncontrolled psychiatric or physical co-morbidity which could, in the opinion of the investigator, compromise participation in this study were not allowed to participate. Family members of the investigational staff, siblings of participants who had also taken part in the previous adult and adolescent study, and any participant with a history of drug/alcohol abuse in the prior 12 months were excluded.

For parent/caregiver interviews, an information sheet was provided outlining the nature of the project to sign and return. For children aged 8–11 years, a story-book assent document was used; whilst children aged 5–7 years were engaged with a verbal study description. For child participants, parental permission was obtained. Once consented, clinicians confirmed eligibility, completed brief medical history forms regarding the child’s UC, and assigned identification numbers to participants.

### Interviews

Interviews were conducted between June 2018 and March 2019. A semi-structured interview guide was developed to guide the discussion and was reviewed by a clinician with expertise in UC and an external consultant with expertise in pediatric qualitative research to verify the clinical relevance and appropriateness of the interview questions. The concept elicitation (CE) part of the interview included open-ended questions to explore patients’ experiences of UC, including symptoms and impact of UC on their daily lives, functioning, and well-being. Initial questions were very broad, allowing concepts to freely arise. The interviewer did not “lead” the discussion, but would probe into pertinent issues raised by the interviewees. If important issues were not discussed spontaneously, then the interviewer utilized more specific probes provided in the interview guide. There was also a short cognitive debrief portion of the interview for some participants (older children and parents/caregivers), but this is not discussed in this manuscript.

The interview duration varied according to the age of the child: it lasted approximately 45 min for children aged 5–7 years and approximately 60 min for the children aged 8–11 years. The interview was longer in older children as this entailed more discussion of symptoms and impacts as their level of cognitive understanding and concentration was better than the younger children. Interviews with parents/caregivers of children aged 2–7 years took approximately 90 min since this had more exploration of how symptoms were observed in this younger cohort. Interviews with parents/caregivers of children aged 8–11 years were focused on symptoms and took approximately 60 min.

All children were asked to develop and bring to their interview a collage representing their UC, though this was a voluntary activity and not required. If a collage was brought, the interviewer asked the participant to explain what each image represented, which allowed this information to facilitate coding [28]. A second creative task involved asking all child participants to think of an animal best representing their UC and probing their choice. This was included to identify which aspect/s of their UC the child may see as most salient and allow further exploration of it in the interview. The value of such creative tasks to qualitative research has been shown to be particularly marked when interviewing younger participants in order to prompt discussion and orient the participant to think about key areas of concern relating to their disease ahead of the interview [28].

### Sample size and saturation

Sampling approaches for qualitative research are driven by a desire to understand the what and why rather than how many. Thus, the sampling approach for this study was “purposive,” meaning that the aim was to recruit “information-rich” individuals from whom the researchers could learn extensively about the issues under examination [29]. As well as being purposive, the sampling technique used allowed for the identification of individuals from a relatively narrow or homogenous sample group (ie, had to have a diagnosis of UC) and, using pre-specified recruitment targets, who cut across age, gender and disease severity. In terms of sample size, the aim was to recruit 15 children and 30 parents/caregivers. This target was based on a previous UC study conducted by the authors [23] and is in line with published literature pertaining to ideal sample sizes for qualitative research [30, 31] where the aim of the research is to achieve data saturation. Although the target study sample was considered sufficient for data saturation, interviews would continue until saturation was met as per current scientific recommendations [32]. Saturation was analyzed for children and parents/caregivers separately. To determine if saturation was met in this study, participants were divided into 3 equal sets based on the chronological order in which they were interviewed [30]. Saturation was evaluated to confirm that the most important symptoms and impact concepts were identified in the interviews and no new concepts would arise with further data collection. Saturation was considered to have been met when no new concepts were discussed in the last set of interviews within each age group [30].

### Analytical approach

All interviews were audio-recorded and transcribed verbatim by a professional transcription company into a Microsoft® Word document that was used for analysis. Inductive thematic analysis [33] was undertaken by 1 expert qualitative researcher. The coding was then reviewed and confirmed by another expert qualitative researcher to ensure codes were being applied consistently. Interpretation was based on theoretical understanding of the disease area and was discussed by the study team. The 6 steps to thematic analysis [33] were followed and, though these may appear linear, this is a flexible and reflective process:

1. Familiarization – reading and re-reading the data and noting initial ideas;
2. Generating codes – coding interesting features systematically;
3. Identifying themes – collating codes into potential themes;
4. Reviewing themes – checking the themes work in relation to the coding and data;
5. Defining themes – refining the themes to make them specific and clear;
6. Report production – selecting clear, vivid examples relating to the research questions in a scholarly report.

After developing an idea of potential relationships between preliminary themes, the next phase of analysis required identification and refinement of any master themes and sub-themes [33]. Themes deemed to be poorly supported in the data were either merged to make stronger themes or identified as not being strong enough to carry forward. All coding was reviewed and discussed by the study team and saturation checked.

For data generated via the animal task, thematic analysis [33] was applied in line with all other qualitative data analysis. The animal task was designed to facilitate spontaneous discussion around participants’ most important experiences of their UC and to generate insight into the nature of UC from the patients’ perspective. Thus, the critical part is not the specific animal chosen, but concepts and experiences represented by the selected animal. All qualitative data were interpreted to understand the experience of participants living with UC in totality, as well as to explore any difference between the 2 child age groups who were asked this question (5–7 years and 8–11 years).

### Ethics

All study documents were submitted and approved by the US Copernicus Group Independent Review Board® on 26 April 2018 (approval number COS1–18-173). This study was conducted in compliance with Good Clinical Practice Guidelines, including International Conference on Harmonization Guidelines [34]. In addition, all local laws and regulatory requirements were adhered to throughout the study.

## Results

### Sample demographics

A total of 32 participants were recruited, consisting of 12 children and 20 parents/caregivers. This consisted of 8 parent/caregiver only interviews and 12 matched dyad interviews (where the child and their parent/caregiver took part in separate interviews).

Challenges with recruitment meant that it was not possible to meet all recruitment targets for disease severity, especially regarding children with severe UC severity because they are typically treated quickly, and it is difficult to identify children with active disease. Sample demographics are presented in Table 1.

**Table 1:** Participant demographics

	Child’s Age 2–4 Years (*n* = 8)	Child’s Age 5–7 Years (*n* = 3)	Child’s Age 8–11 Years (*n* = 9)	Total Child Sample (*N* = 20)
**Child data**				
**Gender**				
Female	2 (25.00%)	0	5 (55.56%)	7 (35.00%)
Male	6 (75.00%)	3 (10.00%)	4 (44.44%)	13 (65.00%)
**Race**				
White	5 (62.50%)	1 (33.33%)	6 (66.67%)	12 (60.00%)
Black/African-American	3 (37.50%)	1 (33.33%)	2 (22.22%)	6 (30.00%)
Asian	0	1 (33.33%)	0	1 (5.00%)
Other	0	0	1 (11.11%)	1 (5.00%)
**Ethnicity**				
Hispanic/Latino	0	0	1 (11.11%)	1 (5.00%)
Not Hispanic/Latino	8 (100.00%)	3 (100.00%)	8 (88.89%)	19 (95.00%)
**Clinician reported severity**				
Mild	6 (75.00%)	1 (33.33%)	4 (44.44%)	11 (55.00%)
Moderate	1 (12.50%)	1 (33.33%)	5 (55.56%)	7 (35.00%)
Severe	1 (12.50%)	1 (33.33%)	0	2 (10.00%)
**Mean months since UC diagnosis**	16.3 (6.88)	21.0 (8.19)	41.7 (20.87)	28.4 (19.03)
**Mean hospitalizations due to UC over past 6 months**	0	0	1 (11.11%)	1 (5.00%)
**Parent/caregiver data**				
**Gender**				
Female	7 (87.50%)	3 (100.00%)	9 (100.00%)	19 (95.00%)
Male	1 (12.50%)	0	0	1 (5.00%)
**Race**				
White/Caucasian	5 (62.50%)	1 (33.33%)	6 (66.67%)	12 (60.00%)
Black/African-American	3 (37.50%)	1 (33.33%)	2 (22.22%)	6 (30.00%)
White/Caucasian	0	1 (33.33%)	0	1 (5.00%)
Other	0	0	1 (11.11%)	1 (5.00%)
**Ethnicity**				
Hispanic/Latino	0	0	1 (11.11%)	1 (5.00%)
Not Hispanic/Latino	8 (100.00%)	3 (100.00%)	8 (88.89%)	19 (95.00%)

Of the 12 child participants, 3 were aged between 5 and 7 years, all of whom were male, and 9 were aged 8–11 years, of whom 5 were female and 4 were male. The mean (standard deviation [SD]) number of months since diagnosis was 28.4 (SD 19.0) and the longest time period since diagnosis was in the 8–11-year-old cohort (41.7 [SD20.1] months). The range of UC severity represented was from mild to severe. Within this range, the 5–7 years matched dyad cohort had 2 children with mild severity, 2 moderate, and 1 severe; the 8–11 years matched dyad cohort had 3 mild, 4 moderate, and 3 severe; the parent/caregiver only cohort (2–4 years) had 3 mild, 4 moderate, and 3 severe. Of the 20 children directly or indirectly involved in the study, only 1 of those aged 8–11 years had been hospitalized due to their UC in the past 6 months.

### Identification of themes and development of a patient-centered conceptual model

**Fig. 1:**
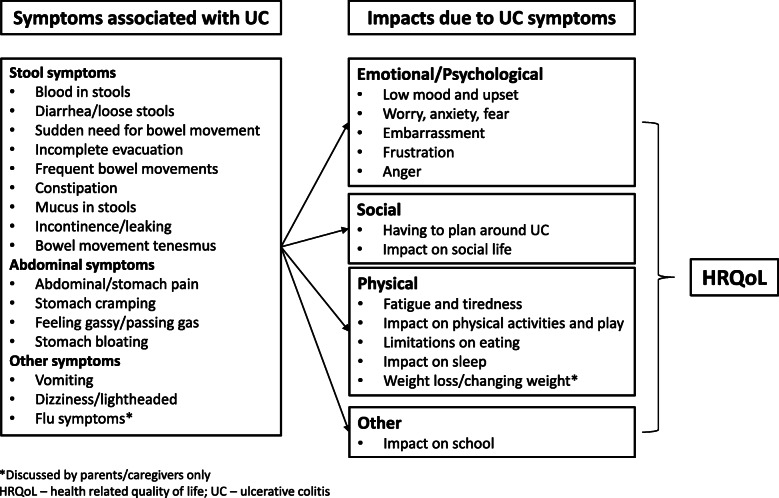
Conceptual Model

Analysis of qualitative data from the interviews revealed a substantial burden of UC on children aged2–11 years old, which added a considerable impact on HRQoL. Following the qualitative analysis of the interviews, the conceptual model was created to include all concepts identified in the interviews (Fig. 1). Several themes relating to symptoms and impacts were identified as important, based on participants’ description of their or their child’s experience and level of agreement within children-caregiver dyads. The figure presents an overall representation of the symptoms and impacts relevant to children aged 2–11 years old with UC, rather than explicitly indicating causation and relationships. Concepts only discussed by parents/caregivers are also indicated in the model. Of note, analysis of concepts by sex within each age group was not possible due to small sample sizes for each age group and the uneven number of male versus female participants within each cohort (35% female in the child cohort and 95% female in the parent/caregiver cohort). Thus, concepts specific to male or female UC patients are not identified in the conceptual model.

Symptoms could be grouped into stool, general abdominal, and other symptoms; whilst impacts could be grouped as emotional/psychological, social, physical, and other. Of note is the fact that impacts of the child’s UC on behavior and HRQoL were less frequently discussed than symptoms.

Table 2 presents all the symptoms and impacts discussed by participants and includes quotes to explore the language used by parents/caregivers and children to describe each concept. Generally, the terms used by parents and children were similar, although child responses were often more descriptive, focusing on events and actions. Participant language was used in the generation of concepts. However, for one concept “BM anticipation/tenesmus,” scientific language was used to develop the concept name due to participants’ quotes being too long and descriptive to be suitable for use as a concept header.

**Table 2:** Participant language when describing each concept

		Parent Example Language	N (%)	Child Example Language	N (%)
**Symptom**	Abdominal/ stomach pain	*“[tells me] stomach’s hurting”* CH-001-F-P [9 Y/O])	20 (100%)	*“tummy hurts*” (RP-013-M-C [5 Y/O])	12 (100%)
	Diarrhea/Loose stool	“*diarrhea comes, it comes fast*” CH-016-F-P (6 Y/O)	20 (100%)	*“[my stool] it’s liquid*” (CH-013-M-C [9 Y/O])	10 (83%)
	Blood in stool	*“blood in his stool*” (CH-008-F-P [4 Y/O])	18 (90%)	“*red blood in the toilet*” (CH-001-F-C [9 Y/O])	11 (91%)
	Stool urgency	*“[child says] ‘I have to poop [now*]” (RP-013-F-P [5 Y/O])	17 (85%)	“*I’ll have to, like, run to the bathroom*” CH-015-F-C [11Y/O])	8 (66%)
	Incomplete evacuation	*“[tells me] I have to go again*” (NO-002-F-P [6 Y/O])	17 (85%)	“*I had to use the bathroom again*” (NC-001-F-C [10 Y/O])	8 (66%)
	Stool frequency	*“[he is] constantly going [to the bathroom]*” (CH-016-F-P [6 Y/O])	16 (80%)	“*Maybe it [number of poops] goes to the 30s*” (CH-014-M-C [8 Y/O])	8 (66%)
	Feeling gassy/ passing gas	*“he has gas”* (CH-004-F-P [11 Y/O])	16 (80%)	“*I have like gas constantly*” (CH-004-M-C)	8 (66%)
	Constipation	*“[he says]‘I have to go to the bathroom, but nothing is coming out*” (CH-014-F-P [8 Y/O])	14 (70%)	“*It feels like I have to do it, and I try doing it, do a number two, but, like, I, like, can’t go*” (CH-001-F-C [9Y/O])	7 (58%)
	Stomach cramping	“*He’s like, “I have a little cramping, mommy*”” H-003-F-P [4 Y/O])	14 (70%)	“*I get cramps because of it*” (CH-007-M-C)	7 (58%)
	Incontinence	“*she’s had some accidents*” (CH-005-F-P [4 Y/O])	14 (70%)	“*see a stain in my underwear”* NC-001-F-C [10 Y/O]	5 (41%)
	Bloating	*“her stomach swells up*” (CH-001-F-P [9 Y/O])	10 (50%)	“*feels like my stomach’s full*” (NC-001-F-C [10 Y/O])	5 (41%)
	Mucus in stool	*“[stool] that’s bloody and mucusy*” (RP-014-F-P [4 Y/O])	13 (65%)	“*It looks like snot, kind of*” (NC-001-F-C [10 Y/O])	2 (16%)
	BM anticipation /tenesmus	“*Sometimes he’ll [go] to the bathroom to poop, but doesn’t have to, so he just sits there*” (CH-004-F-P [11 Y/O])	4 (20%)	*“‘I need to go to the bathroom.’ I go to the bathroom straight away and then ... Then I’m super confused why it doesn’t happen. I’m like, ‘Wait. Why is it not happening?*’” (CH-014-M-C [8 Y/O])	3 (24%)
	Vomiting	*“vomiting”* (CH-004-F-P [11 Y/O])	3 (15%)	*“I threw up”* (RP-013-M-C [5 Y/O])	1 (8%)
	Dizziness/lightheadedness	“*she really feels dizzy*” (NO-001-F-P [11 Y/O])	2 (10%)	“*dizzy*” (N0–001-F-C [11 Y/O])	1 (8%)
	Flu-like symptoms	“*you know, feels kinda sick*” (NO-001-F-P [11 Y/O])	3 (15%)	–	0 (—)
**Emotional/ psychological impacts**	Low mood/upset	“*it [UC] makes him sad***”** (CH-010-M-P [4Y/O])	11 (55%)	“*it [UC] makes me cry*” (CH-001-F-C [9 Y/O])	8 (66%)
	Worry, anxiety, fear	*“he’s afraid”* (NO-002-F-P [6 Y/O])	10 (50%)	*“it scared me”* (CH-013-M-C [9 Y/O])	4 (33%)
	Embarrassment	“*he’s just embarrassed*” (CH-003-F-P [4 Y/O])	10 (50%)	“*it’s kind of embarrassing to have it [UC]*” (CH-007-M-C)	1 (8%)
	Frustration	“*it’s more frustrating with him*” (CH-010-M-P [4 Y/O])	9 (45%)	“*it’s [UC] annoying*” (NC-001-F-C)	1 (8%)
	Anger	*“he get angry at*” (CH-010-M-P [4 Y/O])	1 (5%)	“*I’m angry that my stomach hurts*” (CH-001-F-C [9 Y/O])	1 (8%)
**Physical impacts**	Impact on play & physical activities	“*you know he doesn’t wanna play with anything*” (CH-003-F-P [4 Y/O])	16 (80%)	“*it hurts when I try running*” (CH-001-F-C [9 Y/O])	6 (49%)
	Fatigue and tiredness	“*he would be tired too. Very tired, ‘cause I think it [UC] was just exhausting*” (CH-013-F-P [9 Y/O])	14 (70%)	“*I feel really weak … And lazy … Even though I feel those things*,” (NC-001-F-C [10 Y/O])	3 (24%)
	Limitations on eating	*“loss of appetite”* (CH-013-F-P [9 Y/O]) “[*biggest impact] not being able, being able to eat certain stuff*” (NO-001-F-P [11 Y/O])	11 (55%)	*“[there are] foods I’m not allowed to eat*” (CH-002-F-C [8 Y/O])	3 (24%)
	Impact on sleep	“*he’s gonna be getting up during the night because he has to use the bathroom*” (RP-013-F-P [5 Y/O])	2 (10%)	*“The pain wakes me up”* (CH-015-F-C [11 Y/O])	4 (33%)
	Weight loss or changing weight	“*he lost a lot of weight*” (NO-002-F-P [6 Y/O])	4 (20%)	–	0 (—)
**Social impacts**	Impact on social life	“*There’s many weeks he can’t go see his friends*” (RP-013-F-P [5 Y/O])	5 (25%)	“*I couldn’t go to my church [to see friends*]” (RP-013-M-C [5 Y/O])	3 (24%)
	Having to plan around UC	“*doesn’t wanna be too far away from the bathroom*” (CH-004-F-P [11 Y/O])	2 (10%)	“*I have to like stop and take my medicine*” (NC-001-F-C [10 Y/O])	1 (8%)
**Other**	Impact on school	“*He doesn’t even want to go to school*” (CH-009-F-P [4 Y/O])	14 (70%)	“*Like, I miss some stuff in class*” (CH-004-M-C [11 Y/O])	9 (74%)

The one symptom that was discussed by all 20 parents/caregivers and 12 children was abdominal/stomach pain. When describing this symptom, most parents/caregivers and children used the terms “pain” and “hurting.” In addition, other descriptors were used: “*a tummy ache*” (RP-014-F-P [4 Y/O]); 1 child asserted, “*It feels like someone’s, like, hitting your stomach. And it’s, like, burning pain. Like your stomach is, like, burning. And it feels like just rocks are in there*” (CH-015-F-C [11 Y/O]). Children referred to being poked or stabbed, “*like a needle going into you”* (CH-007-M-C [11 Y/O])*;* 1 parent/caregiver (CH-001-F-P [9 Y/O]) referred to their child describing the pain as if something is moving around inside her, “*She just holds her stomach tells me* ‘*My stomach’s hurting feels like something moving around in my stomach*’” (CH-001-F-P [9 Y/O]).

Symptoms relating to stools (blood in stool, diarrhea/loose stools, stool urgency, incomplete evacuation, stool frequency, and feeing gassy/passing gas) were central for children (regardless of age) and parents/caregivers alike. Some symptoms were associated with higher levels of anxiety. Loose stools, diarrhea, urgency and blood in stools “*most of what comes out of him is blood*” (NO-002-F-P[6 Y/O]) all worsened during flares and were regular symptoms.

Stool urgency was associated with anxiety and “accidents,” it affected the child and the family’s confidence and sense of control, arising “*out of nowhere*” (CH-015-F-C[11 Y/O]) and causing agitation, “*You know, hurry fast*” (CH-003-F-P [4 Y/O]). Frequent bowel movements, bowel movement anticipation, and problems with incomplete evacuation, “*there’s more coming out*” (CH-014-M-C [8 Y/O]) created dependence on washroom availability and, at times, really limited attendance at public venues due to the lack of available toileting facilities. Constipation, sometimes severe, was mentioned less frequently than diarrhea/loose stools and appeared to be less important to participants, but again undermined confidence and control. At the other end of the continuum, incontinence was distressing, embarrassing, and important to participants, especially in the school context. Parents/caregivers suggested their child is “*real conscious about that*” (CH-009-F-P [4 Y/O]). “Accidents” were referred to by several parents/caregivers and their children were often exposed at school by the smell.

During the analysis of the data, some consideration was given to “acute” symptoms that happened only during a UC flare and ongoing “chronic” symptoms that happened between and during UC attacks. In all, 12 out of the total 16 symptoms were categorized as both chronic and acute and would worsen during a UC attack. Whilst vomiting, dizziness/lightheadedness, and flu-like symptoms appeared to happen only during flares, with a “*perfect storm*” of UC symptoms.

Impact on school, and impact physical activities, and play were each discussed by 69% (*n* = 22) of participants. When looking at the child cohort only, impact on school was the most frequently discussed impact (*n* = 9; 75%). In the parent/caregiver cohort, the most frequently discussed concepts were impact on play and physical activities (80%; *n* = 16), school (70%; *n* = 14), and fatigue (70%; *n* = 14). Impact on weight was the only impact mentioned by parents/caregivers, but not by children. In addition, limitations on eating, and the impact on fatigue and tiredness were discussed by parents/caregivers from all age groups, however, only children in the 5–7-year-old cohort discussed these. Despite these exceptions, the impacts discussed were generally consistent across all age groups.

The physical limitations of UC were regularly discussed by parents/caregivers more than by children. Parents/caregivers discussed how children would be more fatigued or tired due to their UC symptoms, “*We can’t get him to do anything, like if we ask him to change his clothes, put his shoes on, he just won’t get up, like he’ll lay on the couch you know, he’ll say his legs hurt, his stomach hurts or you know, he’s tired”* (NO-002-F-P [6 Y/O]). In addition, parents/caregivers (*n* = 16) and children (*n* = 6) discussed the impact on physical activities and play *“Um, like I said he stopped doing karate because he would pretty much spend the whole time running to the bathroom”* (NO-002-F-P [6 Y/O]).

In regard to emotional and psychological impacts, the main areas seen in this research were low mood and being upset, worry/anxiety/fear, embarrassment, frustration, and anger. The most frequently discussed emotional impact was low mood and being upset which was discussed by 8 children and 11 parents/caregivers. Parents/caregivers commented on the child’s habitual sadness, *“I can tell within his eyes”* (CH-016-F-P [6 Y/O]) and referred to the loss and compromise of ability to participate in certain activities. Children themselves stated their unhappiness, “*it makes me cry ‘cause I don’t like it*” (CH-001-F-C [9 Y/O]). Some cried in their sleep due to UC stomach pain, while others referred to a rather bleak sadness they experienced on hospitalization “*I’m like, sad when I’m in the hospital. I feel sad*.” (CH-013-M-C [9Y/O]). One of the children (CH-004-M-C) indicated a reluctance to talk about their emotional state, though they acknowledged sadness and a sense of isolation.

Many parents/caregivers (*n* = 14) discussed how they/their child would experience UC-related worry, anxiety, or fear. Extreme worry and anxiety tended to be positioned as fear, particularly with regard to symptoms like bleeding, which triggered a sense of panic in some children, “*Grandma, I’m bleeding from my butt*” (CH-009-F-P [4 Y/O]), “*I’m gonna lose a lot of, like, a really lot of blood*” (CH-013-M-C [9 Y/O]) and “*he’s asked me before like what if all his blood comes out and he doesn’t have any left?”*(NO-002-F-P [6 Y/O]). It was clear that such fearful anxiety was a key factor in children missing out on activities, play, or social events, demonstrating strong links between emotional and social impacts. The unpredictability of the UC and the perceived lack of control were important elements in these connections.

Embarrassment was strongly associated with the uncertainty of symptom presentation. It was named more frequently by parents/caregivers (10/20) than by children (1/12), for whom it may have been difficult to specifically identify. “Accidents,” soiling of underwear, and for some children, just having UC in general, felt embarrassing, “*it’s kind of embarrassing to have it [UC]*” (CH-007-M-C). This, in turn, fed various experiences of UC frustration and aggravation, with many descriptors being used (*frustrated, aggravated, crabby, grouchy, cranky, annoyed,* and *irritated*). Pain was a particular cause of aggravation. In only 1 or 2 cases (1 caregiver/1 child), was the intensity of these emotions expressed as anger. The child stated, “*I’m angry that my stomach hurts*” (CH-001-F-C [9 Y/O]), whilst the caregiver highlighted their child’s intensified frustration at having to make repeated trips to the bathroom to have a bowel movement, “*he get angry at, over that*” (CH-010-M-P [4 Y/O]).

Negative consequences of these emotional and psychological sequelae affected the social lives of children and their families. Impacts on physical activities and play (mentioned by 22/32 participants) and the impact on school (22/32 participants) included a disinclination to be directly involved or a desire to be left alone when symptoms of UC were prevalent, “*he’ll just want to lay on the couch*” (RP-013-F-P [5 Y/O]), sometimes resulting in isolation, “*he’s not, like, close to anyone at school because he’s not there*” (NO-002-F-P[6 Y/O]). Concerns about loss of school time and negative impact on engagement with school were prevalent. “Accidents” and embarrassment were mentioned and withdrawal from school seen as a way of avoidance. Missing “*some stuff in class*” (CH-004-M-C [11 Y/O]) was an issue for children with UC, just as absence from work is an issue for adults with UC in a very similar research study [23].

In terms of all impacts, coping mechanisms for the challenges and difficulties were generally not mentioned beyond caregivers’ practical strategies, such as trying to help children maintain hydration and giving protein supplements for weight loss.

#### Understanding the nature of UC

The overwhelming and burdensome nature of UC was a clear and pertinent theme throughout the CE interviews with parents/caregivers and children. This was particularly evident in the animal task.

Several animals chosen reflected the aggressive and unpredictable nature of UC, like the jaguar, which “*rears its ugly head*” (NO-001-F-P [11 Y/O]), some of these also suggesting a predatory quality, eg, tiger and panther, “*Something that sneaks up on you and grabs its prey … Before you even see it coming*” (CH-013-F-P [9 Y/O]). The unrelenting character of UC were also seen in examples of the cheetah and the wolf running, “*for like ever and ever*” (RP-013-M-C [5 Y/O]) and the agitated monkey, “*always scratching and rubbing their stomach*” (CH-016-F-P [6 Y/O]). The concept of toughness and resilience was linked to disease flares in mention of the elephant, “*once it gets moving, you can’t stop it*” (CH-004-F-P [11 Y/O]) and turtle, “*Cause it pokes its head out every so often*” (CH-012-F-P [4Y/O]). Some animal choices demonstrated specific symptoms, such as the cheetah representing diarrhea, “*he runs fast … when the diarrhea comes, it comes fast*” (CH-016-F-P [6 Y/O]), frequency of bowel movements (eg, elephant and bird, “*they poop all the time*” (NC-001-F-C[11 Y/O]) and pain, with the example of the lion, “*Because when she’s in pain, it’s like her stomach roars*” (CH-015-F-P [11 Y/O])). Emotional difficulties associated with UC were also revealed; these included feeling unclean/dirty, like a “*messy*” pig (CH-007-F-P[11Y/O]) and the discomfort of co-existence with UC was well-characterized in the disliked rat and the “*creepy and weird*” (BNC-001-F-C [10 Y/O]) Komodo dragon.

#### Level of agreement between child and parent/caregiver dyads

In terms of the level of agreement between children and their parents/caregivers regarding the presence/absence of symptoms and consequent impact, the information from the 12 dyad interviews (ie, three 5–7-year-old dyads and nine 8–11-year-old dyads) were used and calculations were done based on the number of instances of agreement on the presence/absence of a symptom or area of impact. This is presented in Table 3.

**Table 3:** Frequency counts for agreement on presence/absence of a symptom/impact by the parent and child during dyad interviews

		5–7 Year Olds (*n* = 3)	8–11 Year Olds (*n* = 9)	Total (*N* = 12)
**Symptom**	Abdominal/stomach pain	3	9	12
	Blood in Stool	3	8	11
	Diarrhea/Loose stool	2	8	10
	Feeling gassy/passing gas	1	8	9
	Incomplete evacuation	1	7	8
	Stool frequency	0	8	8
	Stool urgency	2	6	8
	Stomach cramping	3	5	8
	Bloating	2	6	8
	Constipation	2	5	7
	Incontinence	1	4	5
	Mucus in stool	0	5	5
	BM anticipation/tenesmus	0	2	2
	Dizziness/lightheaded	0	1	1
	Vomiting	0	0	0
	Flu like symptoms	0	0	0
**Emotional/ psychological impacts**	Low mood/upset	1	2	3
	Worry, anxiety, fear	0	3	3
	Upset	1	2	3
	Embarrassment	0	0	0
	Frustration/aggravation	0	0	0
	Anger	0	0	0
**Physical impacts**	Impact on physical activities and play	2	4	6
	Fatigue & tiredness	0	2	2
	Limitations on eating	0	1	1
	Impact on sleep	0	0	0
	Weight loss or changing weight	0	0	0
**Social impacts**	Impact on social life	1	0	1
	Having to plan around UC	0	0	0
**Other**	Impact on school	2	4	6

A review of the data shows that abdominal/stomach pain was the only symptom discussed by all children and by all parents/caregivers in all dyads. However, within-dyad agreement was high for other frequently occurring symptoms (ie, presence/absence of blood in stool, diarrhea, and feeling gassy or passing gas). As expected, symptoms that were less frequently discussed had a lower level of agreement; for example, flu-like symptoms were only discussed by parents/caregivers, whilst vomiting was discussed by adults but only 1 child.

Among the younger children, 5–7 years, all children and parents/caregivers also discussed blood in stool and stomach cramping, there was general agreement (> 66%) for stool urgency, diarrhea/loose stools, bloating, and constipation. However, parents/caregivers in this age group were more likely to mention a symptom that was not reported by their child (eg, stool frequency and mucus in stool).

The agreement in the description of symptoms experience was much higher in 8–11-year-olds. There was a high level of agreement (> 75%) for blood in stool, stool frequency, diarrhea, incomplete evacuation, and feeling gassy or passing gas. Constipation, bloating, stool urgency, and cramping all had a moderate level of agreement (> 50%).

Impacts were less frequently discussed by all participants, and, as such, there was also less agreement on these. The highest level of agreement (> 50%) was with impact on school and impact on physical activities and play.

### Saturation

Saturation analysis demonstrated that no new concepts were introduced within the last set of parent/caregiver interviews, and therefore saturation was considered to have been met in the parent/caregiver cohort.

For children, no new symptoms were reported in the last set of interviews, but the impact “having to plan around UC” was mentioned in the last cohort only. However, this impact was only discussed by 1 child aged 5–7 years and no children aged 8–11 years, despite this being discussed by all parents/caregivers. Upon review of the data with the study team and discussion with experts in pediatric research, it was agreed that this was unlikely to be discussed in other child interviews since this is likely something considered by the parents/caregivers only rather than the child in these age groups. Thus, saturation was met for both symptoms and impact concepts in the child cohort.

## Discussion

Qualitative analysis of data from the CE interviews disclosed the substantial burden of UC and its impact. The key symptoms and impacts identified from the qualitative analysis, reflecting the lived experience of UC seen directly through the eyes of the children themselves and indirectly, through those of their parents/caregivers, closely mapped onto those identified in studying both adolescents and adults [23]. This resulted in the generation of a conceptual disease model. With the exception of a few impacts (impact on school versus impact on work), the conceptual model of children’s UC maps onto the conceptual model of adult and adolescent UC developed in an earlier publication [23] and clarifies for us that symptoms and experiences are similar, regardless of age.

Though the young age of some participants proved a challenge for recruitment, analysis of the data contributed by younger children showed experiential homogeneity. Analysis of the qualitative data revealed a substantial burden associated with UC and how its unpredictable and disruptive nature undermines confidence and negatively impacts HRQoL. In addition, the emotional strain of living with UC is clearly evident from these interviews and reflects closely the findings of other similar studies [23, 35–37].

When looking at the animal task there were many similarities between the animals and concepts identified by parents/caregivers and their children. This is indicative of the fact that both children and parents/caregivers are aligned with regards to the nature of the child’s UC.

Most of the symptoms described by participants occurred regularly and were described as worsening during an attack of UC (chronic) whereas others only occurred during a UC flare and so were considered more acute symptoms. In all, 12 out of the 16 symptoms mentioned by participants were described as being present most of the time (chronic) but worsening during an attack of UC. While vomiting, dizziness/lightheadedness, and flu-like symptoms appeared to happen only during flares, where their UC was bad. There was no difference in the experience of these symptoms by age group.

Previous qualitative research has primarily focused on adults, with most studies utilizing patients with mixed inflammatory bowel disease (IBD) etiologies [5–22]. A failure in these studies to analyze data by specific condition, made it previously impossible to differentiate findings attributable to UC versus other IBD conditions. Therefore, building on a recent study exploring the symptomatic and HRQoL impacts of adults and adolescents with UC [23], the current study has substantially added to our understanding of the UC disease journey in childhood.

Some of these physical and emotional impacts identified may have particular significance at the young ages of those in the current study. The particular consequences of early and repeated frustration and embarrassment with out-of-control bodily functions remains little understood.

When looking at HRQoL impacts, impact on school, and impact on physical activities and play were discussed most by participants. Of some interest is the fact that impact on the child’s weight was the only impact mentioned by parents/caregivers but not children, and impact on eating, and the impact on fatigue, and tiredness were discussed by parents/caregivers of all age groups, but generally not by children, with the exception of the 5–7-year-old age cohort.

When taking into account the overall picture and looking at the previous related publication [23], symptomatology and patient journey appear so similar from childhood into adulthood as to effectively be the same. Therefore, what we are aiming to treat in children, adolescents, and adults with UC is both the source of these symptoms that are common to all and the physical, emotional, psychological, social, and physical consequences of living with UC.

## Limitations

It was not possible to identify more children with severe UC (*n* = 3), to meet initial recruitment targets. Following feedback from clinicians, this was due to children with severe UC being treated quickly to reduce their symptoms, therefore, they could no longer be classified as severe.

It must also be noted that participants were recruited from the US only, so care should be taken in assuming that the results are generalizable to other cultures. Nonetheless, support from the literature and a recent similar study [23] with adolescents and adults, as well as clinician feedback, suggests that wider geographic diversity would have been likely to highlight the same concepts.

## Conclusions

Qualitative analysis of data from these CE interviews disclosed the substantial emotional and physical burden of UC, as well as the impact on participants’ lives. The significant symptomatic and HRQoL impacts of living with UC affected children in ways that clearly show homogeneity with previous studies [5–23]. There was a high level of agreement between children and parents/caregivers regarding the presence or absence of symptoms. Children aged 8–11 years demonstrated higher agreement with caregivers than younger children, indicating that symptom self-report in those aged 8–11 years could be reliably used in future studies.

The conceptual model generated as part of this research reflects the lived experiences of this health condition from the patient perspective. It also clearly maps onto the conceptual model developed in the previous study of adults and adolescents [23], demonstrating conceptual continuity and similarity of symptom and HRQoL impact.

## Data Availability

The datasets generated and/or analyzed during the current study are not publicly available due to the sensitive nature of the questions asked in this study, but are available from the corresponding author on reasonable request.

## Abbreviations

C: Child
CE: Concept elicitation
F: Female
FDA: Food and Drug Administration
HRQoL: Health-related quality of life
IBD: Inflammatory bowel disease
O: Old
P: Parent/caregiver
M: Male
PRO: Patient-reported outcome
SD: Standard deviation
UC: Ulcerative colitis
US: United States
Y: Years
